# Regulation of Cell Wall Synthesis by the Clathrin Light Chain Is Essential for Viability in *Schizosaccharomyces pombe*

**DOI:** 10.1371/journal.pone.0071510

**Published:** 2013-08-19

**Authors:** Nagore de León, Mohammad Reza Sharifmoghadam, Marta Hoya, M.-Ángeles Curto, Cristina Doncel, M.-Henar Valdivieso

**Affiliations:** 1 Departamento de Microbiología y Genética/IBFG, Universidad de Salamanca/CSIC, Salamanca, Spain; 2 Department of Biology, Faculty of Science, Ferdowsi University of Mashhad, Mashhad, Iran; University of Cambridge, United Kingdom

## Abstract

The regulation of cell wall synthesis by the clathrin light chain has been addressed. *Schizosaccharomyces pombe clc1Δ* mutant was inviable in the absence of osmotic stabilization; when grown in sorbitol-supplemented medium *clc1Δ* cells grew slowly, formed aggregates, and had strong defects in morphology. Additionally, *clc1Δ* cells exhibited an altered cell wall composition. A mutant that allowed modulating the amount of Clc1p was created to analyze in more detail the dependence of cell wall synthesis on clathrin. A 40% reduction in the amount of Clc1p did not affect acid phosphatase secretion and bulk lipid internalization. Under these conditions, β(1,3)glucan synthase activity and cell wall synthesis were reduced. Also, the delivery of glucan synthases to the cell surface, and the secretion of the Eng1p glucanase were defective. These results suggest that the defects in the cell wall observed in the conditional mutant were due to a defective secretion of enzymes involved in the synthesis/remodelling of this structure, rather than to their endocytosis. Our results show that a reduction in the amount of clathrin that has minor effects on general vesicle trafficking has a strong impact on cell wall synthesis, and suggest that this is the reason for the lethality of *clc1Δ* cells in the absence of osmotic stabilization.

## Introduction

*Schizosaccharomyces pombe* is an attractive model to study cell morphogenesis; this yeast is rod-shaped, grows in a polarized asymmetric way by extension of the ends, and divides by medial fission. *S. pombe* cell shape is determined by the actin cytoskeleton, microtubules, and cell wall. Actin localizes at the cell tips and the cell division site, and changes its distribution throughout the cell cycle. Actin patches participate in the internalization of endocytic vesicles, and are considered markers of cellular polarity [1], [2], [3], [4]. Microtubules are cylindrical polymers of tubulin molecules that contribute to the establishment of cell polarity [2], [5]. The fungal cell wall is a morphogenetic element that determines the final shape of fungal cells and protects them against lysis in hypo-osmotic environments. In *S. pombe*, the cell wall is composed of α- and β-glucans, and mannoproteins [6]. Bgs1/Cps1p, Bgs3p, and Bgs4p are essential β-glucan synthases, and Eng1p and Agn1p are glucanases required for cell separation [7], [8], [9], [10], [11]. The fact that in this fission yeast there is no chitin in the cell wall allows studying the regulation of glucan synthesis without the interference of the compensatory mechanisms that in other fungi trigger chitin synthesis when glucan is defective [12], [13], [14].

Most of the enzymes involved in cell wall biosynthesis are membrane-bound or cell wall-associated proteins that must be delivered to the cell surface in order to exert their function. There is some information about the trafficking of the chitin synthase Chs3p in *S. cerevisiae* [15], [16]; however, almost nothing is known about the regulation of glucan synthesis by the vesicle trafficking mechanisms in any organism. Clathrin plays a major role in the formation of coated vesicles, which mediate protein sorting from the trans-Golgi network to different cellular organelles and clathrin-dependent endocytosis. Three units of clathrin light chain associate with three units of clathrin heavy chain to form triskelions, which assemble to form a lattice that surrounds the clathrin-coated vesicles. The cargo proteins are recognized by diverse adaptor complexes that, in turn, bind the clathrin lattice. In yeast, the AP-1 adaptor complex mediates sorting in the late-Golgi and/or in endosomes; the AP-2 complex associates with clathrin at the plasma membrane to mediate endocytosis, and the AP-3 complex is required for the transport from the Golgi an/or endosomes to the vacuole [17], [18], [3], [4].

In *Saccharomyces cerevisiae*, null mutants for the clathrin heavy-chain *CHC1* gene were obtained in the 1980s [19], [20]; the lethality of the *chc1Δ* mutants was found to depend on the presence of second-site mutations that impaired growth [20], [21]. *chc1* mutants were able to secrete proteins [19], [22] and had slow growth and aberrant morphology [19], [20]. While sequences for clathrin heavy chains are conserved, those of clathrin light chains are more divergent [23], [24]. Although *S. cerevisiae* mutants deleted for either the *CHC1* or *CLC1* genes share many phenotypes, *clc1Δ* mutants are viable in all genetic backgrounds tested [23], [25]. Similarly, in the amoeba *Dictyostelium discoideum* the *chc1Δ* mutant is viable, although cells grow slowly [26], and eliminating the clathrin light chain elicits milder phenotypes than those of *chc1Δ* deletion [24]. Surprisingly, according to genome-wide analyses of gene deletions, *S. pombe clc1Δ* cells are inviable, as well as *chc1Δ* cells [27].

The aim of this study was to gain information about the regulation of cell wall synthesis by the mechanisms of vesicle trafficking; in particular, we were interested in knowing how this process is regulated by clathrin. To fulfil this purpose, we cloned the *S. pombe clc1^+^* gene; surprisingly, it was found that *clc1Δ* cells depended on the presence of an osmotic stabilizer for viability. β(1,3)glucan synthases were mis-sorted and cell wall synthesis was diminished in cells lacking *clc1^+^*. By analyzing a mutant with reduced amounts of Clc1p, we found that cell wall synthesis is one of the most clathrin-dependent processes in fission yeast, a result that helps to explain the dependence of *clc1Δ* mutants on sorbitol for viability.

## Materials and Methods

### Strains and growth conditions

All techniques for *S. pombe* growth and manipulation have been described previously ([28]; www.biotwiki.org/bin/view/Pombe/NurseLabManual; PombeNet: www-bcf.usc.edu/∼forsburg/index.html). The relevant genotypes and source of the strains used are listed in Table S1. Cells were grown in either rich medium (YES), YES supplemented with 1.2 M sorbitol, or minimal medium (MM) with appropriate supplements and incubated at 28°C. G418 (ForMedium) was used at 120 μg/ml. L-Azetidine-2-carboxylic acid (AZC; SIGMA) was used at 0.9 mg/ml.

### Genetic methods

Molecular and genetic manipulations were according to Sambrook *et al*. [29]. A *clc1* null mutant was constructed by transforming a diploid strain with a cassette in which the KANMX6 selection marker was flanked by 1kb-DNA fragments containing the 5′ and 3′ *clc1^+^* untranslated regions. Spores were dissected by micromanipulation in YES and YES plus sorbitol plates. HA-Clc1p was produced by cloning the HA epitope as a *Not*I DNA fragments into a *Not*I site created by mutagenesis after the initiation codon of *clc1^+^*; the tagged protein was functional according to its capacity to support growth of a *clc1Δ* null mutant in medium without sorbitol. A 41X*HAclc1*:KAN allele was produced using the N-terminal protein-tagging procedure described by Bähler *et al*. [30]. A diploid strain was transformed with the PCR-amplified cassette and transformants were selected on PMG (MM in which the NH_4_Cl had been replaced by L-glutamic acid) plates supplemented with sorbitol and 300 μg/ml G418. Tetrads were dissected on PMG with and without sorbitol. The AP-2 complex subunit Apl3p (ORF SPBC691.03c) was tagged with GFP at its C-terminal end using a *Not*I DNA fragment containing the fluorescent protein; Apl3p was separated from the fluorescent protein by a hinge of 12 alanines to avoid distortion of the protein. All tagged proteins were integrated into the chromosome under the control of their endogenous promoters. Combination of the 41X*HAclc1*:KAN mutant allele with tagged proteins was performed by random spore germination [28] on PMG with G418 plates and selection of clones by PCR and fluorescence microscopy analyses.

### Acid phosphatase measurements

To determine acid phosphatase secretion, the enzymatic activity in the culture medium and in cell extracts was assessed. Cells were grown exponentially in MM with or without phosphate and sorbitol at 28°C. To assess activity in the culture medium, 3×10^6^ cells from each culture were collected by centrifugation. 500 μl of the supernatant was combined with 500 μl of substrate solution (2 mM p-nitrophenyl phosphate, 0.1 M sodium acetate, pH 4.0) pre-warmed to 30°C, and incubated at 30°C for 5 min. Reactions were stopped by the addition of 500 μl of 1M sodium hydroxide. The absorbance at 405 nm was measured. The results were expressed relative to the number of cells and considered arbitrary units. To determine the enzymatic activity in cell extracts, a larger volume of cells from the same culture was collected by centrifugation; cells were washed twice with water and once with 50 mM sodium acetate, pH 4.0. Cells were broken in the presence of 50 mM sodium acetate, pH 4.0, and protease inhibitors, as described [31]. 5 μl from each extract was added to 495 μl of 50 mM sodium acetate, pH 4.0. This mixture was combined with 500 μl of substrate solution, and the acid phosphatase activity was measured as explained for the culture medium. The activity, as arbitrary units, was estimated per number of cells in the culture, so the values could be compared with those obtained for the activity in the culture medium. The secreted activity was estimated as units in the medium with respect to the total units (units in the medium plus units in the cells). Each experiment was performed a minimum of three times.

### Cell wall analysis

β glucan synthase activity and cell wall composition were analyzed as described [7], [31].

### Microscopy

Calcofluor (0.125 μg/ml) and Hoechst 33258 (50 μg/ml) staining were performed as described [31], [32]. Actin staining was performed using rhodamine-phalloidin and Alexa Fluor 488-phalloidin. FM4-64 (Biotium) internalization was performed as follows: cells were kept on ice for 2 minutes, combined with the dye (at a final concentration of 25 μM from a stock at 10 mM in water) and photographed immediately. In order to detect the vacuoles, the cells were incubated at 32°C in the presence of the dye for 30 minutes.

For fluorescence microscopy, images were captured with either a Leica DM RXA conventional microscope equipped with a Photometrics Sensys CCD camera, using the Qfish 2.3 program, or with an Olympus IX71 microscope equipped with a personal DeltaVision system and a Photometrics CoolSnap HQ2monochrome camera. In the latter case, stacks of z-series sections were acquired at 0.2-μm intervals. Unless stated otherwise, fluorescence images are maximum two-dimensional projections of the 6 z-series that corresponded to the middle of the cell and were analyzed using deconvolution software from Applied Precision. Images were processed with Adobe Photoshop, IMAGEJ (National Institutes of Health), or SOFTWORX DV software.

Fluorescence Recovery After Photobleaching (FRAP) experiments were performed using a OLYMPUS IX 81 spinning disc microscope with a PLan Apo 100x/1.4 Oil objective, equipped with a CSUX1-A1 Confocal Head (Yokogawa) and a EVOLVE form PHOTOMETRICS camera. FRAP was performed with an iLas, laser line 491 module (ROPER Scientific). The data were obtained and analyzed with Metamorph software. The whole cell pole was irreversibly bleached by a short laser pulse of appropriate wavelength and the recovery of fluorescence signal as a function of time was measured. Images were acquired before photobleaching, immediately after, and subsequently at regular intervals. Data were plotted with Graphpad Prism software.

Electron microscopy was performed on cells fixed in glutaraldehyde (EM grade, SIGMA) and stained with permanganate, as described [33]. Ultrathin sections were cut on a Jung Reichert microtome (Leica Mikroskopie and System GmbH) and examined using a JEM1010 transmission electron microscope (Jeol) at 100 kV.

### Protein analyses

Western blot was performed as described [31] using anti-GFP (JL8, BD Living Colors; 1∶500), anti-HA (12CA5; Roche, 1∶4000), anti-Cdc2 p34 (Y100.4; Santa Cruz Biotechnology, 1∶5000), anti-α tubulin (clone B-5-1-2; SIGMA, 1∶10000), and anti-Cpy1 (A6428; Invitrogen, 1∶100) primary antibodies and anti-mouse horseradish peroxidase-conjugated secondary antibody (BIORAD, 1∶10000).

To detect Eng1-GFP protein, 50-ml cultures (about 5×10^8^ cells) were collected by centrifugation at different times after the addition of thiamine. The culture medium was concentrated to 200 μl using Amicon Ultra-15 centrifugal filter devices (Millipore) and boiled in a final volume of 400 μl in the presence of Laemmli sample buffer (50 mM HCl-Tris, pH 6.8; 1% SDS; 143 mM β-mercaptoethanol; 10% glycerol). From the pellets, cell extracts were obtained as described ([31]; in this protocol cell walls are discarded with cell debris) and boiled in a final volume of 400 μl of Laemmli sample buffer. 40 μl from each sample (cytosol and culture medium) was loaded into 6.5% polyacrylamide gels. Thus, for each time-point the amount of sample from the medium and the cytosol came from the same volume of the original culture, and could therefore be compared directly.

### Statistical analyses

The SPSS Statistic 17.0 software was used to evaluate statistically the results performing Student's t-test; the results were validated with the Wilcoxon–Mann–Whitney test where indicated. Values significantly different from the controls are indicated.

## Results

### *clc1Δ* mutants depend on osmotic stabilization for viability

**Figure 1:**
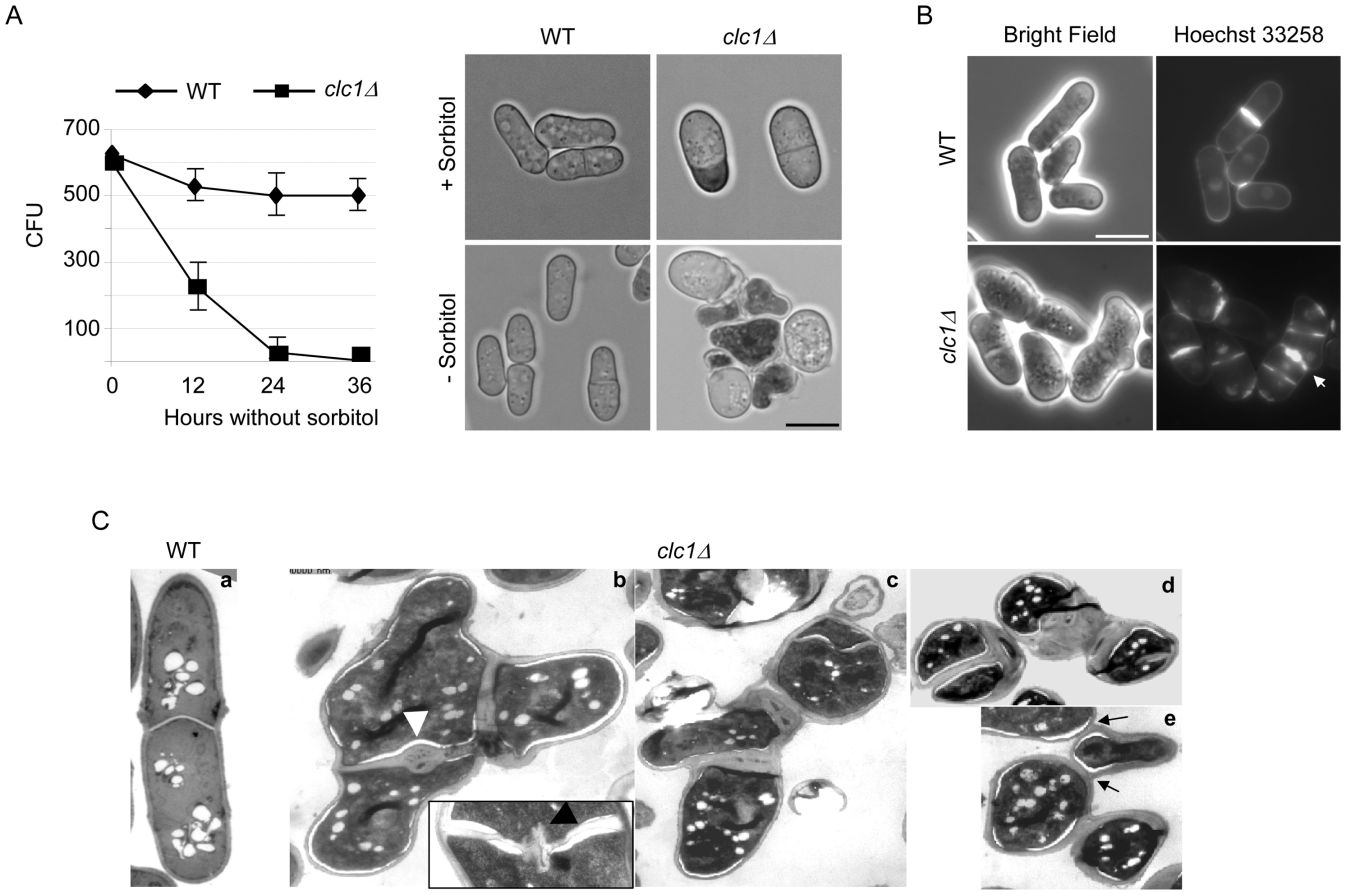
*clc1Δ* cells are inviable in the absence of sorbitol and exhibit defects in morphogenesis. A. Left panel, cells from the indicated strains were transferred from YES plus sorbitol to YES medium and incubated at 28°C. At the indicated times, 600 cells were plated onto sorbitol-supplemented YES medium and incubated at 28°C for five days; the colony-forming units (CFU) were scored. The experiment was performed three times; the means of the values and the standard deviations are represented. Right panel, cells from the indicated strains grown in the presence or the absence of sorbitol for 12 hours were stained with Methylene blue and photographed. B. Cells from the wild-type and *clc1Δ* strains were grown in YES with sorbitol and stained with Hoechst 33258 to observe nuclei and cell wall; the arrow denotes a septum thickened in its central region. In A and B the bar represents 10 μm. C. Electron microscopy of wild-type and *clc1Δ* cells grown in the presence of sorbitol. One cell from the control strain (panel *a*) and different cells from the null *clc1Δ* mutant (panels *b* to *e*) are shown. Arrowheads in panel *b* denote septa that are thickened at their central region; arrows in panel *e* point to cell wall remnants that join cells after division.

In order to generate a *clc1Δ* mutant, a diploid strain was transformed with a *clc1::KANMX6* cassette and tetrads were dissected on YES plates. Only two spores produced colonies and these colonies were sensitive to geneticin, confirming that *clc1Δ* mutants are inviable, in agreement with the information obtained in systematic deletion analyses [27]. Spore germination is a challenging process that requires the action of cell wall-degrading enzymes, which can produce cell lysis; this effect can be reduced by adding an osmotic stabilizer to the dissection plates. When tetrads from the *clc1^+^*/*clc1::KANMX6* diploid were dissected on YES medium supplemented with sorbitol, four spores formed colonies, two of which grew on YES plates supplemented with sorbitol and geneticin (not shown). In order to determine whether the requirement of *clc1Δ* cells for osmotic stabilization was restricted to spore germination, cells were grown in YES with sorbitol and transferred to sorbitol-free medium. Aliquots were withdrawn at different times and 600 cells were plated onto YES plus sorbitol plates. Analysis of the colony-forming units revealed that 12 hours after the removal of the osmotic stabilizer, viability was reduced to about 30%, and that most cells were dead after 24 hours (figure 1 A, left panel). The time-point 0 hours corresponds to cells that were transferred from YES plus sorbitol liquid medium to YES plus sorbitol plates. Methylene blue-staining showed that about 50% of the cells were lysed after 12 hours of incubation in YES without sorbitol (figure 1 A, right panel). When the *clc1Δ* mutant was transformed with an integrative plasmid bearing an HA-tagged Clc1 protein cells were able to grow in the absence of sorbitol, showing that lethality was due to the lack of *clc1^+^*, not to second-site mutations. Thus, *clc1Δ* cells required osmotic stabilization for growth. All the experiments involving the *clc1Δ* mutant described below were performed incubating the mutant and control cells in the presence of sorbitol.

### *clc1Δ* cells have strong defects in cell wall synthesis

The fact that cells required an osmotic stabilizer for growth strongly suggested that the *clc1Δ* strain had a defect in cell wall synthesis. Additionally, *clc1Δ* cells exhibited an aberrant morphology; they were rounder and often larger than the control cells, defects that are often observed in cell wall mutants. When cells were stained with Hoechst 33258, which allows simultaneous observation of cell walls and nuclei, differences in nuclear staining between the wild-type and mutant cells were not observed. Regarding the cell wall, in the wild-type strain the fluorescence was distributed uniformly along the cell surface and was strong at the septa, while in the mutant strain the cell surface appeared discontinuous and most septa (over 90% of closed septa; a minimum of 100 septa were scored in each of three independent experiments) were abnormally thick at the central region (see septa denoted by an arrow in figure 1 B). Additionally, more than 50% of the cells had more than one septum. This abnormal distribution of the cell wall was confirmed using the cell wall-specific dye Calcofluor (not shown). In order to analyze the cell wall in greater detail, we observed wild-type and mutant cells by electron microscopy. This approach confirmed that the absence of Clc1p led to dramatic defects in cell wall distribution, which included a lateral wall that was irregular in its thickness and septa that were swollen or deformed at their central region (arrowheads in figure 1 C, panel b). Multiseptated/branched cells were also present and it was also possible to observe cells that remained joined after cell division even in the absence of deformed septa (see arrows in figure 1 C, panel e).

**Figure 2:**
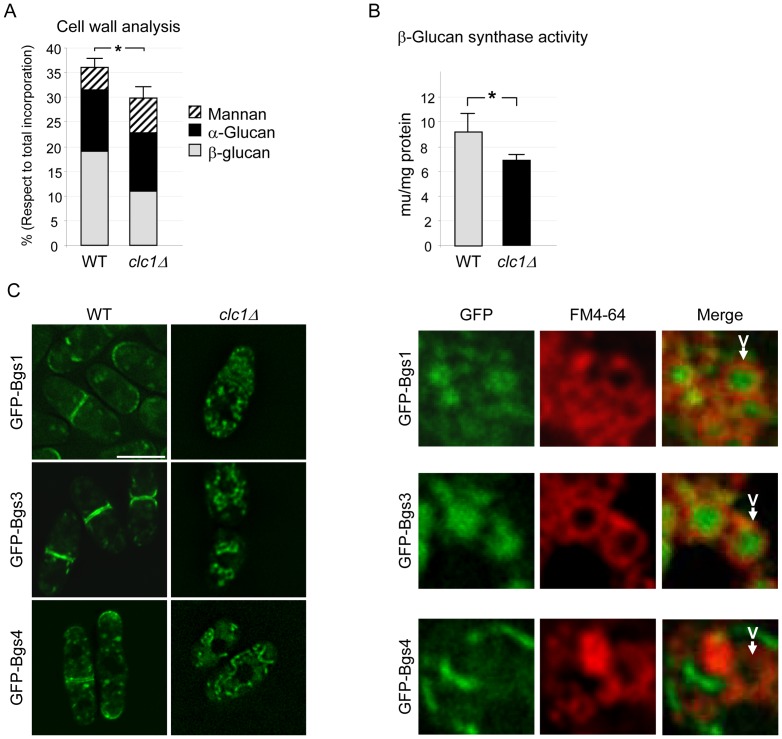
Cell wall synthesis is defective in *clc1Δ* cells. A. Cell wall analysis of wild-type and *clc1Δ* cells. The graph represents the percentage of incorporation of radioactivity into the cell wall polysaccharides of cultures grown in YES with sorbitol in the presence of ^14^C-glucose. The experiment was performed a minimum of five times with duplicates. For the total glucose incorporation into the cell wall, the standard deviation and statistical significance of the difference (*, p<0.05. Student's t-test) is shown. B. β(1,3)glucan synthase activity in wild-type and *clc1Δ* cells incubated in YES with sorbitol. The experiment was performed a minimum of five times with duplicates; the means, standard deviations, and statistical significance of the difference (*, p<0.001. Student's t-test) are shown. C. Localization of the β(1,3)glucan synthases Bgs1p, Bgs3p, and Bgs4p in the wild-type and *clc1Δ* strains (medial sections) incubated in YES with sorbitol. Right panels, vacuoles in *clc1Δ* cells were counterstained with FM4-64. V, vacuole. Bar, 10 μm.

In order to characterize better the defect in cell wall synthesis in the *clc1Δ* mutant, we analyzed the cell wall composition in wild-type and mutant cells (figure 2 A). The incorporation of ^14^C-glucose into the cell wall with respect to the total incorporation into cellular material was significantly reduced in the *clc1Δ* strain (36% for the wild-type and 29% for the mutant). Upon analyzing the ^14^C-glucose incorporated into each of the cell wall polymers, we found that there was a reduction in β(1,3)glucan synthesis in the mutant (19.0±2.0% and 11.1±1.5% for the wild-type and *clc1Δ* strains, respectively); α-glucan synthesis was similar in both strains (12.4±1.6% and 11.7±1.4% for wild-type and *clc1Δ*, respectively) and mannan synthesis was increased in the mutant (4.6±0.7% and 7.1±0.9% for the wild-type and mutant strains, respectively). Thus, in the *clc1Δ* strain cell wall composition was defective, the synthesis of β(1,3)glucan and mannoproteins being more affected than the synthesis of α-glucan. *In vitro* measurements of β(1,3)glucan synthase activity showed that the value for the *clc1Δ* cells was 80% of the wild-type control (figure 2 B). The experiment was performed five times with duplicates; the value for the mutant cells was always lower than the value for the control). These defects in β-glucan synthesis measured *in vivo*, and in β-glucan synthase activity measured *in vitro* are stronger than those detected in some *bgs1/cps1* and *bgs4* glucan synthase mutants [7], [10].

To obtain further information about the role of clathrin in β(1,3)glucan synthesis, we analyzed the distribution of the GFP-Bgs1p, GFP-Bgs3p and GFP-Bgs4p β(1,3)glucan synthases in the absence of Clc1p. In the control strains, all three enzymes were detected at the cell growth areas (cell poles and equator) and in internal vesicles; in the *clc1Δ* mutant, none of the enzymes was detected at the cell surface (figure 2 C, left panels). According to counter-staining with FM4-64, GFP-Bgs1 and GFP-Bgs3 were mis-sorted to the vacuoles, while GFP-Bgs4 was detected in some tubular structures that did not correspond to vacuoles. In sum, the absence of Clc1p alters the trafficking of the three essential β(1,3)glucan synthase enzymes leading to a defective cell wall synthesis, which contributes to the aberrant cell morphology and the requirement for osmotic stabilization of *clc1Δ* cells. The defect in the distribution of GFP-Bgs1 was more severe than that described for the *apm1Δ* mutant [34], in agreement with the fact that clathrin is required for AP-1-independent trafficking events.

### Construction of a mutant with reduced levels of Clc1p

**Figure 3:**
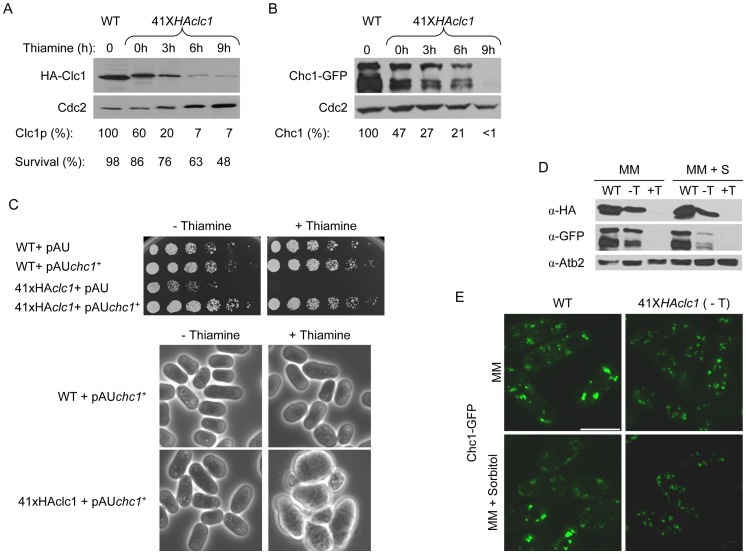
Characterization of clathrin in the 41X*HAclc1* mutant. A. Western blot of extracts from cells in which the *clc1^+^* gene, fused to the HA epitope, was driven from its native promoter or from the 41X*nmt1^+^* promoter and were incubated in the presence of thiamine (repressing conditions) for the indicated hours (h). The amount of Clc1p was calculated considering the amount of protein produced from the *clc1^+^* promoter as 100%. To calculate cell survival, samples were withdrawn from the culture at the indicated times; 600 cells were plated onto YES with sorbitol, and the number of colony-forming units was scored. The experiment was performed three times. The data correspond to the mean. B. The same as in A but the cells carried a Chc1-GFP fusion protein. C. Upper panel, 3×10^4^ cells and serial 1∶4 dilutions from the indicated strains were inoculated on minimal medium minus uracil, without and with thiamine, and incubated for three days at 28°C. Lower panes, cells from the indicated strains and conditions were taken from the plates and photographed using a conventional phase-contrast microscope. D. Western blot of extracts from the wild-type strain and the 41X*HAclc1* mutant bearing HA-Clc1 and Chc1-GFP fusion proteins. Cells were grown in minimal medium and minimal medium with sorbitol in the absence (−T) and the presence (+T) of thiamine for 15 hours. α-Atb2 (tubulin) was used as a loading control. E. Micrographs of wild-type and 41X*HAclc1* cells bearing Chc1-GFP grown in minimal medium and minimal medium with sorbitol in the absence of thiamine. Micrographs (projections) were taken with a DeltaVision deconvolution microscope. Bar, 10 μm.

As described above, the *clc1Δ* cells had a highly-penetrant phenotype. The cells had aberrant morphology, formed clumps, and grew slowly. Additionally, these cells mated very poorly and their efficiency of transformation was extremely low (likely because of cell wall defects and fragility), which precluded a more detailed analysis of Clc1p function. Finally, all the experiments had to be performed in the presence of sorbitol, which induces stress responses [35] and affects endocytosis [36], [37]. In order to overcome these problems, we constructed a strain in which the amount of Clc1p could be modulated; this mutant would allow us to identify those cellular processes that are most sensitive to a clathrin deficiency. To construct such a strain, the *clc1^+^* ORF fused to the HA epitope at the N-terminal end of the protein was expressed under the control of the medium-strength 41X*nmt1^+^* promoter, which is repressible by thiamine; replacement of the promoter and protein tagging took place at the *clc1^+^ locus*. Western blot analyses were performed to estimate the level of protein in cells grown in the absence of thiamine or in the presence of the vitamin for different times. As a control, a strain in which the *HA-clc1^+^* allele was integrated into the chromosome and expressed from its endogenous promoter was used. As shown in figure 3 A, even in the absence of thiamine (promoter de-repressed) the amount of Clc1p was reduced to about 60% in the mutant strain with respect to the control. After 3 hours of repression, the amount of Clc1p was 20% that of the control strain, and this amount was less than 10% after 6 hours of repression. The viability of the mutant was 86±6%, 76±4%, 63±5%, and 48±6% (colony-forming units with respect to inoculated cells) after 0, 3, 6, and 9 hours of growth in the presence of thiamine, respectively; the experiment was performed three times. After longer incubation times in the presence of thiamine, viability was greatly reduced and the morphology of cells was aberrant (not shown). Under these conditions, 41X*HAclc1* cells could be kept alive in sorbitol-supplemented medium and exhibited a phenotype similar to that of the null *clc1Δ* mutant (not shown).

We next analyzed whether the clathrin heavy chain was affected by a reduced level of Clc1p. As shown in 3 B, Chc1-GFP was detected as a series of bands in the wild-type and mutant strains; the relative intensity of each band varied from experiment to experiment. In the mutant strain, the amount of Chc1p was lower than that of the control strain when the amount of Clc1p was reduced; after nine hours of *clc1^+^* repression, Chc1-GFP was not detected. These results show that Clc1p is required for the stability of Chc1p and support the idea that the phenotypes found in the 41X*HAclc1* mutant are due to defects in clathrin. To confirm this hypothesis, a wild-type strain and the 41X*HAclc1* mutant were transformed with the pAU multicopy vector and with the same vector carrying the *chc1^+^* gene. When the cells were incubated in the absence of thiamine (de-repressing conditions for the conditional mutant; small reduction in the amount of Clc1p), the multicopy plasmid bearing *chc1^+^* was able to suppress the mild growth defects of the 41X*HAclc1* mutant (figure 3 C). Additionally, the morphology of the cells was similar to that of the control cells. In the presence of thiamine (repressing conditions), the plasmid was able to support growth of the 41X*HAclc1* mutant. Under these conditions, the morphology of the cells resembled that of the conditional mutant grown in the presence of thiamine and sorbitol for long incubation times. Thus, increasing the amount of Chc1p suppressed the phenotypes produced by a small reduction in the amount of Clc1p. However, when Clc1p was absent, Chc1p was not completely functional. These results are in agreement with those found in *S. cerevisiae*, where Clc1p is required for the full functionality of clathrin so that overexpression of *CHC1* only suppresses some of the phenotypes of a *clc1* mutant [25], [38].

**Figure 4:**
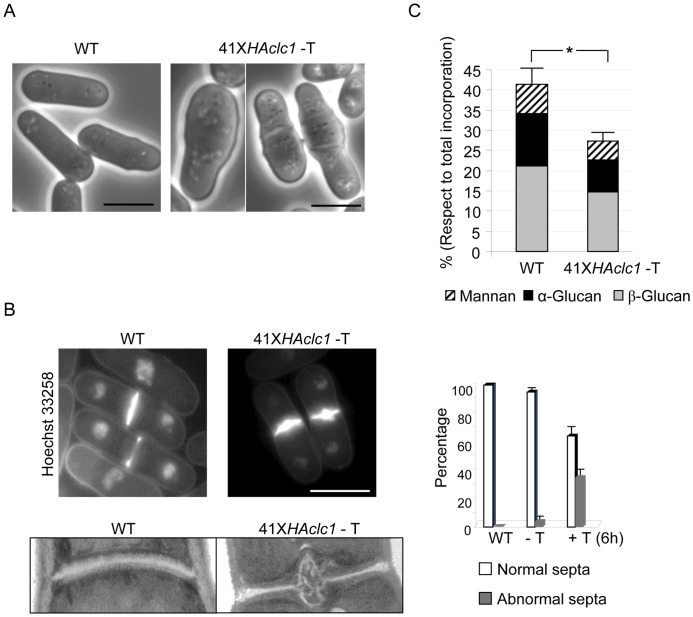
Morphology and cell wall synthesis are abnormal in 41X*HAclc1* cells. A. Phase-contrast microscopy of wild-type and 41X*HAclc1* cells grown in the absence of thiamine. B. Upper left panel, Hoechst 33258 staining of cells from the control and mutant strains grown in the absence of thiamine. Right panel, quantification of normal and abnormal septa in wild-type and mutant cells grown in the absence or the presence of thiamine for six hours; a minimum of 100 septa were scored in each of three independent experiments. Lower panel, electron microscopy of septa from the indicated strains. C. Cell wall analysis of wild-type and 41X*HAclc1* cells. The graph represents the percentage of incorporation of radioactivity into the cell wall polysaccharides of cultures grown in the absence of thiamine and the presence of ^14^C-glucose. The experiment was performed five times with duplicates. The standard deviation and statistical significance (*, p<0.01. Student's t-test) for the total incorporation in the cell wall is shown.

**Figure 5:**
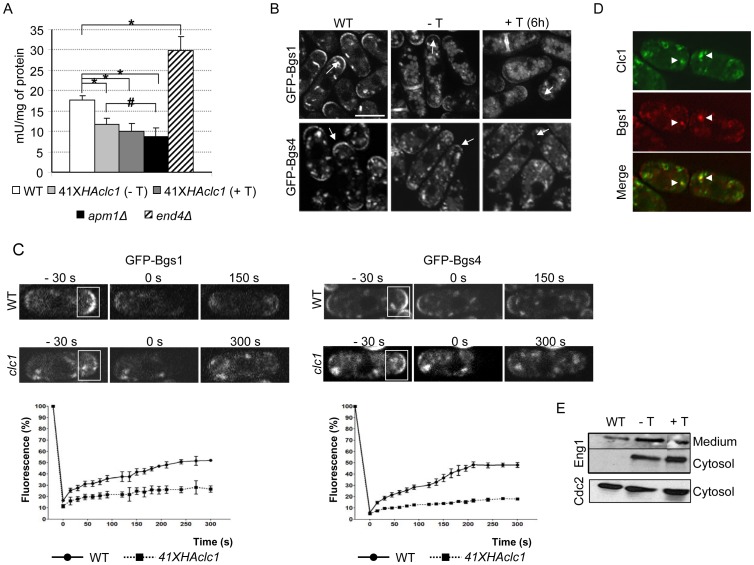
Secretion of enzymes required for cell wall synthesis is defective in the 41X*HAclc1* mutant. A. β(1,3)glucan synthase activity in the indicated strains grown in minimal medium without thiamine, except for the 41X*HAclc1* (+T) sample, that was incubated with the vitamin for 6 hours. The experiment was performed three times with duplicates; the mean values, standard deviations, and statistical significance (*; p<0,0001. #; p<0.01. Student's t-test, confirmed by Wilcoxon-Mann-Whitney test) are represented. B. Distribution of GFP-Bgs1 and GFP-Bgs4 in cells from the wild-type and 41X*HAclc1* cells grown with or without thiamine for six hours. Arrows denote cell poles. Images are projections of z-stacks captured with a DeltaVision deconvolution system. Bar, 10 μm. C. Cells expressing GFP-Bgs1 and GFP-Bgs4 were photo-bleached at time 0 in the indicated regions and then imaged over time (upper panels). The lower panels show the quantification of the recovery of fluorescence intensity (percentage of the initial value for each strain). Each plot represents the average value for a minimum of 7 cells from three experiments. Error bars show the standard error of the mean. D. Co-localization between Clc1-GFP and RFP-Bgs1. Arrowheads denote Golgi/endosomal structures where both proteins co-localize. E. Western blot analysis of Eng1-GFP in the wild-type and 41X*HAclc*1cells incubated in the absence or the presence of thiamine for six hours. The amount of protein from the culture medium or cytosol came from the same volume of the original culture and can therefore be compared directly. Cdc2 in the cytosol is shown as a loading control.

**Figure 6:**
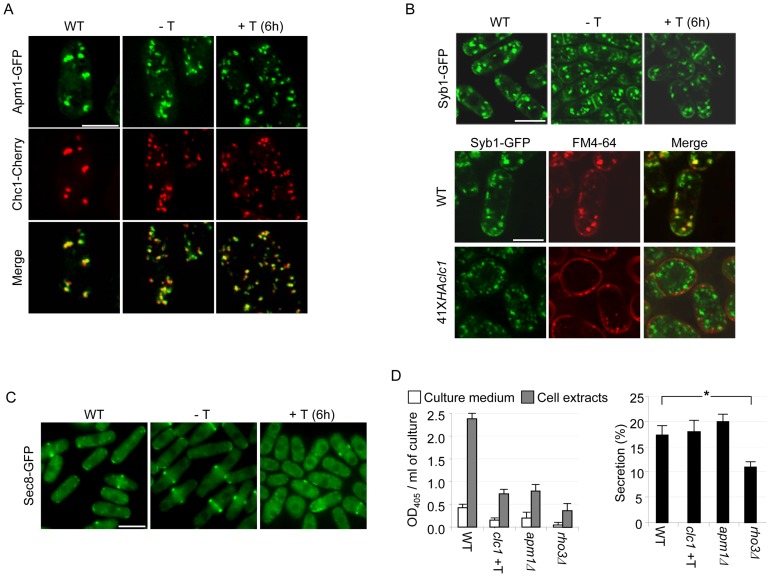
Analysis of general secretion in 41X*HAclc1* cells. A. Micrographs of wild-type (WT) and 41X*HAclc1* cells bearing Apm1-GFP and Chc1-mCherry fusion proteins; cells were incubated in the absence (−T) and the presence (+T) of thiamine for 6 hours. The micrographs are projections of z-stack images taken with a DeltaVision deconvolution microscope. B. Upper panels, micrographs of wild-type (WT) and 41X*HAclc1* cells bearing Syb1-GFP incubated in minimal medium in the absence (−T) and the presence (+T) of thiamine for 6 hours. The micrographs are medial sections. Lower panels, the same as before but the cells were incubated in the presence of thiamine and sorbitol for 48 hours. C. Micrographs of the indicated strains carrying a Sec8-GFP fusion protein; the photographs were taken with a conventional fluorescence microscope. Bars, 10 μm. D. Acid phosphatase activity was measured in the culture medium and cell extracts as indicated in Materials and methods; all samples were collected from logarithmic cultures at a density of 2×10^7^ cells per ml. Left panel, activity as arbitrary units in the culture medium and cell extracts from the indicated strains grown in the presence of thiamine for 6 hours. The activity in the cell extracts was estimated per number of cells in the same volume of culture that was used to estimate activity in the medium, so that both data can be compared directly. Right panel, the secretion of acid phosphatase was calculated as the percentage of activity in the culture medium with respect to the total activity detected (activity in the medium plus activity in cell extracts). The experiment was performed a minimum of five times with duplicates; statistically significant defects are indicated (*; p<0.05; Student's t-test).

**Figure 7:**
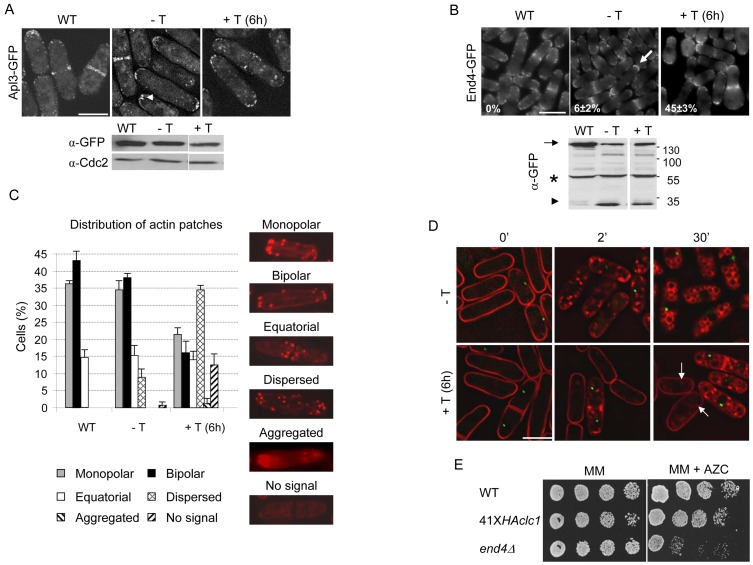
Analysis of endocytosis in 41X*HAclc1* cells. A. Upper panels, Apl3-GFP distribution in the wild-type (WT) and 41X*HAclc1* strains grown in the absence (−T) and the presence of thiamine (+T) for 6 hours; the arrowhead denotes clustered Apl3-GFP dots. Lower panel, Anti-GFP and anti-Cdc2 Western blot from the same samples. B. Same as in A but the cells bear End4/Sla2-GFP. The arrow denotes a cell that exhibits a continuous fluorescence at the cell surface. The number of cells with such a distribution of End4-GFP is indicated for each sample. Lower panel, Western blot analysis of cell extracts from the same samples. The arrow denotes End4-GFP, the arrowhead denotes a band that could correspond to the GFP, and the asterisk marks an unspecific band. C. Quantification of the number of cells with different actin patch distribution with respect to the total cell number. Right panels, pictures showing the patterns of actin patch distribution that were scored; cells were stained with Rhodamine-Phalloidin. The experiment was performed three times; a minimum of 500 cells were scored in each experiment. D. FM4-64 uptake in mixed cultures of wild-type cells bearing the spindle-pole-body Sad1 protein fused to the GFP and 41X*HAclc1* cells grown in the absence and the presence of thiamine for six hours. Cells were stained with the dye and photographed after the indicated times (in minutes) of incubation at 32°C; arrows denote internalized FM4-64. The fluorescence images were obtained with a DeltaVision deconvolution microscope; in A, B, and C the images are projections of z-stacks, and in D are medial sections. The bar represents 10 μm. E. 3×10^4^ cells and serial 1∶4 dilutions from the indicated strains were spotted onto MM plates and MM plates supplemented with 0.9 μM of the L-proline analogue L-azetidine-2-carboxylic acid (AZC) and incubated at 28°C for five days before being photographed.

Thus, cells growing in the absence of thiamine are mutants partially defective in clathrin; comparing their phenotype with that of the control strain should allow detecting the most clathrin-dependent cellular processes. Growth in the presence of thiamine for different times represents a more restrictive condition that would allow studying how a further reduction in the amount of Clc1p affected these processes, and how new cellular processes were affected. Unless stated otherwise, in the experiments described below the analysis of phenotypes was performed using the control strain (HA-*clc1^+^* under the control of its native promoter in figure 3, and untagged *clc1^+^* in figures 4–7), and the 41X*HAclc1* strain grown in the absence of thiamine (de-repressing conditions; mild reduction in the amount of Clc1p) and in the presence of the vitamin for 6 hours (repressing conditions; strong reduction in the amount of Clc1p).

Finally, we investigated whether the suppression of lethality in *clc1^+^*-depleted cells by sorbitol was due to an increase in the amount of Chc1p produced by osmotic stabilization. Western blot analyses showed that the amount of Chc1-GFP decreased in the 41X*HAclc1* strain when thiamine was added either to minimal medium or to minimal medium with sorbitol (figure 3 D). The amount of Chc1-GFP was lower when the mutant strain was grown in minimal medium with sorbitol than when it was grown in minimal medium; we do not know either the reason for this effect of sorbitol or its physiological relevance. In a wild-type strain, Chc1-GFP was observed as a series of fluorescent patches into the cells ([39]. Figure 3 E). In the 41X*HAclc1* mutant grown in the absence of thiamine, Chc1-GFP was also observed as a series of intracellular dots, although these dots were slightly smaller than those of the control strain (figure 3E; see also Chc1-Cherry in figure 6A), in agreement with the reduced amount of Chc1p detected by Western blot in the mutant strain (figure 3 B). As shown in figure 3 E, the presence of sorbitol did not produce any alteration in the distribution of Chc1-GFP. Thus, the suppression of lethality in *clc1^+^*-depleted cells by sorbitol was not due to an increase in the amount of Chc1-GFP produced by osmotic stabilization.

### Analysis of cell wall in cells with a reduced amount of Clc1p

Observation of 41X*HAclc1* cells growing actively under de-repressing conditions showed that 15% of the cells had an aberrant morphology, with swollen tips or central region (two examples are shown in figure 4 A); usually, these cells were bigger than those of the control strain. This number was 23% after 6 hours of growth in the presence of thiamine. This phenotype was indicative of an abnormal growth pattern and/or a weakened cell wall. Additionally, cell staining with Hoechst 33258 revealed that a small number of cells (4%) had septa that were thickened in their central region (figure 4 B); this number was 35% after six hours of repression (figure 4 B right panel; a minimum of 100 septa were scored in each of three independent experiments.). Electron microscopy confirmed that some septa had an aberrant structure (figure 4 B, lower panel).

In order to characterize in more detail the cell wall synthesis process in cells with a reduced amount of clathrin, cell wall composition analyses were performed in the wild-type and mutant cells grown in the absence of thiamine; cell wall synthesis was reduced to 64% in the mutant, with respect to the control strain (figure 4 C). This reduction affected the three cell wall polymers. Since the conditional mutant was grown in minimal medium while the null mutant was grown in rich medium with sorbitol, the absolute values for cell wall content cannot be compared directly.

### Analysis of glucan synthesis in cells with a reduced amount of Clc1p

To gain further information about the nature of the regulation of cell wall synthesis by clathrin, we analyzed the effect that a disturbance in protein trafficking had on the β(1,3)glucan synthase activity measured *in vitro*. The activity in the 41X*HAclc1* mutant grown under de-repressing and repressing conditions was compared to that of the wild type strain, to that of the *apm1Δ* mutant (defective in exocytosis, [40]), and to that of the *end4Δ* mutant (defective in endocytosis, [41]). The experiment was performed three times with duplicates; in all the experiments, the tendency of the values was the same. The mean value for the activity in the 41X*HAclc1* mutant was 66% that of the control strain when grown under de-repressing conditions (figure 5 A). In the *apm1Δ* mutant, the activity was 59% that of the control strain while in the *end4Δ* mutant the activity was 170% that of the control. Thus, the level of activity in the *clc1* conditional mutant was more similar to that of the mutant defective in exocytosis than to that of the mutant defective in endocytosis. Additionally, a further reduction in the amount of clathrin by adding thiamine to the culture decreased the activity in the mutant to a 56% of the value for the control strain. After 6 hours of repression, the reduction in the activity was mild with respect to the activity under de-repressing conditions, while the phenotypes were significantly enhanced. This apparent contradiction can be explained by the fact that the *in vitro* experiment was carried out for a short incubation time (30 minutes). In the *in vivo* experiments the cells have been growing with a reduced level of β(1,3)glucan synthase activity for six hours. Additionally, before adding thiamine to the cultures the glucan synthase activity was significantly reduced in the mutant with respect to the wild-type strain. Thus, the aggravation of the phenotypes after repression would have been produced by the accumulation defects along time.

Recently, a reduction in the localization of Bgs1p at the cell poles has been described in the *apm1Δ* null mutant [34]. This mutant exhibits multiple defects in different cellular processes [40]; therefore, it is difficult to determine whether the reduced distribution of Bgs1p at the cell poles in this strain is directly produced by the absence of its secretion or it is an indirect effect of the alteration in some other cellular process. Bgs1p is the β(1,3)glucan synthase responsible for the synthesis of the linear β(1,3)glucan, which is detected by Calcofluor staining at the cell septum and poles [42], but its contribution to the activity detected *in vitro* is minimal [7], [10]. Bgs4p participates in septation and is the β(1,3)glucan synthase responsible for most of the activity detected *in vitro* [7], [10]. Accordingly, we analyzed their distribution in the control and mutant strain in the absence and presence of thiamine. The fluorescent signal corresponding to both enzymes was weaker at the cell poles in the mutant strain than in the control strain, even under de-repressing conditions (figure 5 B). Conversely, the mutant cells exhibited more GFP-Bgs1p and GFP-Bgs4p fluorescence inside the cells. These results suggested that when the level of clathrin is reduced, Bgs1p and Bgs4p are mis-sorted. Thus, our results confirm those of Yu *et al*. [34] and expand them showing that the regulation of cell wall synthesis by clathrin is not restricted to Bgs1p.

To confirm the hypothesis that in the 41X*HAclc1* mutant secretion of the Bgs enzymes was diminished, FRAP experiments were performed. In this kind of experiment, the kinetics and the level of recovery of fluorescence in the bleached area of the cell are analyzed; usually, the recovery of fluorescence in the bleached area is below 100%, even in the control situation. In our case, cell poles in wild-type and 41X*HAclc1* mutant cells bearing either GFP-Bgs1 or GFP-Bgs4 grown in minimal medium without thiamine were bleached completely to avoid lateral diffusion of the fluorescence. Three independent experiments were performed; for each experiment a minimum of seven cells were analyzed. According to computer-assisted measurements, the fluorescence of GFP-Bgs1 and GFP-Bgs4 recovered with a half-time (t_1/2_) of 81 and 73 seconds, respectively, in the wild-type strain. For both GFP-Bgs1 and GFP-Bgs4, the recovery of fluorescence at the cell poles in the 41X*HAclc1* mutant strain was less efficient than in the control strain (figure 5 C). In the mutant strain, some fluorescence recovered in the region of the cytoplasm that had been bleached; however, no fluorescence was recovered at the surface of the cell poles even 300 seconds after the bleaching (figure 5 C). These results show a slow turnover of the Bgs enzymes in the mutant strain, supporting the idea of a defective transport of the proteins towards the cell surface.

In agreement with clathrin being involved in the trafficking of Bgs proteins, we found *in vivo* co-localization between Clc1-GFP and RFP-Bgs1 at Golgi/endosomes (figure 5 D).

Finally, we wished to investigate whether the regulation of cell wall synthesis by clathrin was restricted to the control of β(1,3)glucan synthases. To address this question, we analyzed the effect of reducing the amount of clathrin in the secretion of the endo-β(1,3)glucanase Eng1p, which is required for cell separation; Eng1p is synthesized in the cytoplasm and needs to be secreted to degrade the septum from the outside of the cell [8]. As shown in figure 5 E, in the wild-type strain this protein was only detected in the culture medium while in the mutant strain the protein was detected in the cytosol and culture medium even under de-repressing conditions. The presence of the protein in the medium was not due to cell lysis since, according to Methylene blue staining, there was no lysis in the wild-type or mutant strains under these culture conditions (not shown). Thus, the rate of Eng1 secretion is decreased when clathrin is reduced. All these results strongly suggest that the defects in cell wall synthesis detected in the 41X*HAclc1*strain grown under de-repressing conditions were primarily due to defects in secretion, rather to defects in endocytosis.

In summary, a 40% reduction in the amount of Clc1p (the reduction in this protein in the 41X*HAclc1* cells grown under de-repressing conditions) has a significant effect on cell wall composition. This defect is due to a defective secretion of enzymes required for the synthesis and remodelling of this essential structure. Although cell wall synthesis was reduced, the defective structure synthesized by cells under de-repressing conditions was able to support cell integrity, since cells were viable and did not lyse in the absence of an osmotic stabilizer.

### Analysis of general secretion in cells with reduced levels of Clc1p

It was possible that the defects in cell wall synthesis observed in the 41X*HAclc1* mutant were the consequence of a general defect in vesicle trafficking in this strain. To analyze this possibility, vesicle transport was characterized in this strain by several approaches. Initially, we used fluorescence microscopy to analyze the distribution of proteins involved in secretion. The AP-1 complex subunit Apm1-GFP was observed as cytoplasmic dots, most of which co-localized with Chc1-mCherry (figure 6 A). In agreement with a reduction in the amount of Chc1p, in the mutant strain the fluorescent dots were smaller than in the control; however, even after 6 hours of *clc1^+^* repression Chc1p and Apm1p still co-localized. Thus, a reduction in Clc1p apparently did not eliminate Chc1p ability to be recruited to membranes with Apm1p. Similarly, we did not find any apparent difference between the distribution of the v-SNARE (vesicle soluble N-ethylmaleimide-sensitive factor attachment protein receptor) Syb1p in the wild-type and the mutant strains grown in the absence or the presence of thiamine for six hours (figure 6 B, upper panels). It has been described that the loss of the AP-1 complex perturbs the distribution of Syb1-GFP so that this SNARE is not observed at the cell surface in an *apm1Δ* strain [40]; in the 41X*HAclc1* mutant this phenotype was only observed when cells were incubated under repressing conditions for 48 hours (in sorbitol-supplemented medium. Figure 6 B, lower panels), a condition equivalent to the *clc1Δ* deletion. This result is in agreement with our observation that in the 41X*HAclc1* strain the β(1,3)glucan synthase activity is higher than in the *apm1Δ* mutant, even after six hours of repression. Finally, we analyzed the distribution of the exocyst protein Sec8-GFP. This protein localized to the cell poles and midzone in the control strain and in the 41X*HAclc1* mutant incubated in the absence of thiamine; after 6 hours of *clc1^+^* repression, about 50% of the cells exhibited Sec8-GFP that was not localized to the cell growth zones but dispersed throughout the cytoplasm (figure 6 C).

Next, we used a biochemical approach to analyze secretion in the 41X*HAclc1* mutant. It has been described that invertase and acid phosphatase are secreted at almost wild-type levels in the *S. cerevisiae* and the *D. discoideum chc1Δ* mutants [19], [22], [26], while this activity was greatly reduced *S. pombe apm1Δ* mutants [40]. Acid phosphatase is a highly regulated activity that depends on the medium composition and growth conditions [43], [44]. Therefore, a possible explanation for this apparent discrepancy might be that in the former cases the activities were measured in the cells and culture medium while in the latter the activity was only assayed in the medium. When we measured acid phosphatase activity in the culture medium we found that it was reduced in the *apm1Δ*, *rho3Δ,* and 41X*HAclc1*mutants (in the presence and absence of thiamine. Figure 6 D, left panel, and results not shown). Similar results were obtained when the activity was measured in cell extracts (figure 6 D, left panel). When the proportion between the activity detected in the medium and the total activity (in medium and cell extracts) was calculated, we found no significant differences between the wild-type and 41X*HAclc1* cells, even under repressing conditions (figure 6 D, right panel); using this approach we found a secretion defect in the *rho3Δ* mutant, defective in a Rho-type GTPase involved in secretion [45], but not in the *apm1Δ* mutant. A similar result was obtained when the activity was measured in *clc1Δ* cells incubated in the presence of sorbitol (figure S1).

Mis-sorting of the vacuolar carboxypeptidase Y to the cell surface is frequently used to asses a defective transport between the Golgi and the vacuoles. Dot-blot analyses performed to detect mis-sorted Cpy1p to the cell surface in the 41X*HAclc1* strain under de-repressing conditions gave a negative result (figure S2). The experiment could not be performed at 6 hours of repression because the plates had to be incubated for several days.

All these results show that a 40% reduction in the amount of Clc1p (the amount of protein estimated in mutant cells growing in the absence of thiamine) produce minor alterations in the secretory machinery that do not lead to a general defect in protein secretion. A further reduction in the amount of Clc1p is accompanied by additional alterations in the secretion machinery.

### Analysis of endocytosis in cells with reduced levels of Clc1p

Clathrin is required for endocytosis; therefore, this process was analyzed in the 41X*HAclc1* mutant. The AP-2 complex subunit Apl3p was observed as a series of small dots located close to or at the cell surface of the cell poles and equatorial area in the control strain (figure 7 A). When the amount of Clc1p was reduced, cells exhibited fewer Apl3-GFP dots, which in a few cells (less than 10%; a minimum of 100 cells in each of three different experiments were scored) were larger than those of the control strain and/or formed clusters (see arrowhead in the panel −T of figure 7 A). The dots were mostly observed at the cell surface, suggesting that either their mobility was reduced or their lifetime at the plasma membrane was affected by the reduction in Clc1p. According to Western blot analysis, the amount of Apl3-GFP was slightly reduced in the 41X*HAclc1* mutant. With respect to End4/Sla2-GFP, an adaptor protein that links actin to clathrin and is necessary for endocytosis [41], [46], in the wild-type strain this protein was observed as a series of dots located either at or just beneath the cell surface along the cell body, being concentrated at cell poles and midzone (figure 7 B). In the 41X*HAclc1* mutant grown in the absence of thiamine, about 6% of cells exhibited a continuous End4-GFP signal that was exclusively concentrated at the cell surface of the cell growth areas (see the arrow in figure 7 B; a minimum of 100 cells in each of three different experiments were scored). This percentage increased when thiamine was added to the cultures, reaching 45% after 6 hours of repression. According to Western blot analyses, the total amount of End4-GFP was not significantly reduced in the mutant; however, the protein was cleaved when the cells had a reduced amount of Clc1p, since a band that could correspond to the GFP fragment of the End4-GFP fusion protein was observed in the mutant but not in the control strain. In *S. cerevisiae* Clc1p binds Sla2/End4p and promotes the progression the Sla2p patches at the endocytic sites so that Clc1p positively regulates Sla2p for efficient endocytosis [47], [48]. The reduced amount of clathrin in the *S. pombe* 41X*HAclc1* mutant probably slows the progression of the End4p patches at the endocytic sites and renders End4-GFP unstable. Actin patches mark the sites of active endocytosis and participate in this process [3], [4]; in the 41X*HAclc1* mutant cultured under de-repressing conditions, 9.5% of the cells exhibited disperse (depolarized) actin patches and 1% of the cells had no patches. After 6 hours of repression, there was a dramatic modification in the distribution of actin patches, 34% of the cells exhibiting dispersed patches; 13% of the cells with no patches, and 1.2% of the cells with aggregated patches.

The effect of those alterations in the endocytic machinery on bulk lipid endocytosis was assessed by observing FM4-64 uptake in mixed cultures from a control strain bearing the spindle pole body protein Sad1-GFP and the 41X*HAclc1* strain. The cultures were incubated in the absence or the presence of thiamine; photographs were taken 0, 2, and 30 minutes after the addition of the dye (figure 7 D. The experiment was performed three times with similar results). In the absence of thiamine, FM4-64 was observed at the cell surface at the 0-minute time-point, in internal endosomes after 2 minutes, and in the vacuoles after 30 minutes in the wild-type and mutant strains (cells bearing and lacking the green Sad1-GFP dots, respectively). When cells were incubated for 6 hours in the presence of thiamine, FM4-64 stained the cell surface of both strains at the beginning of the experiment (0′); after 2 minutes, the dye was observed inside the wild-type cells (marked by the green Sad1-GFP dots) but not inside the mutant cells (those lacking the GFP dot); after 30 minutes of incubation, FM4-64 was observed in the vacuoles of the control strain. Under these conditions, the dye was still detected at the cell surface in the mutant cells; only a weak staining was observed inside some cells (arrows in the corresponding panel of figure 7 D). Thus, a 40% reduction in the amount of Clc1p did not affect FM4-64 uptake, and a 90% reduction in this protein delayed this process, but did not abrogate it.

The endocytosis of some plasma membrane transporters was assessed by analyzing growth in the presence of L-azetidine-2-carboxylic acid (AZC), a toxic analogue of L-proline that becomes incorporated into proteins; this drug inhibits cell growth in the *end4/sla2Δ* mutant due to a defect in the endocytosis of the corresponding amino acid transporter ([41] and figure 7 E). We did not find any differences in the growth of the wild-type and the 41X*HAclc1* mutant in the absence of thiamine (figure 7 E). The experiment cannot be performed in minimal medium with thiamine because 41X*HAclc1* cells die before 24 hours of incubation in the presence of the vitamin.

In summary, a 40% reduction of Clc1p (the reduction in the 41X*HAclc1* cells grown under de-repressing conditions) produced some alterations in the endocytic machinery that did not lead to a general defect in endocytosis. A further reduction in the amount of Clc1p produced a significant alteration in the process.

## Discussion

### The *S. pombe* clathrin light chain

In this work, the impact of decreasing the amount of Clc1p in the fission yeast *S. pombe* has been examined. *clc1Δ* cells were unable to grow unless osmotic stabilization was provided; when grown in sorbitol-supplemented medium, *clc1Δ* cells exhibited slow growth, aberrant morphology, increased size, and sexual development defects. Similar phenotypes have been observed in *S. cerevisiae* and *D. discoideum clc1Δ* and *chc1Δ* mutants, although in these organisms *clc1Δ* mutants are viable in all the conditions tested and deleting *clc1^+^* is less deleterious than deleting *chc1^+^*. This different requirement for clathrin light chain could be explained by the fact that in *D. discoideum* eliminating the light chain has no effect in the abundance of the clathrin heavy chain [24]. In *S. cerevisiae*, deletion of *CLC1* leads to a decrease in the amount of Chc1p; the triskelions formed under these conditions are unstable but still partially functional, as shown by the fact that *CHC1* overexpression complements some defects in the *clc1Δ* mutants [25], [38]. In *S. pombe*, Chc1-GFP could not be detected by Western blot after 9 hours of *clc1^+^* repression, suggesting that in the fission yeast Clc1p is essential for the stability of Chc1p. These results strongly suggest that the phenotypes found in the *S. pombe clc1* mutants are due to defects in clathrin. This idea is supported by the fact that overexpression of *chc1* ^+^ suppresses the defects of the 41X*HAclc1* mutant incubated under de-repressing conditions.

### Cell wall synthesis and viability in *clc1* mutants

Another relevant question is why the depletion of clathrin is more detrimental for *S. pombe* than for *S. cerevisiae*, where *chc1Δ* and *chc1Δ clc1Δ* deletions are only lethal in the presence of mutations in other *loci* that compromise cell growth [20], [21], and for the amoeba *D. discoideum*, where the *chc1Δ* mutant is viable [26]. A possible answer could lie in the cell wall composition in these organisms. *D. discoideum* does not have a cell wall during vegetative growth. In *S. cerevisiae*, the cell wall has β(1,3)glucan and chitin, and it is known that defects in glucan synthesis triggers compensatory mechanisms that increase the amount of chitin to allow viability [12], [13], [14]. *S. pombe* has no detectable amounts of chitin [49], [50] and hence defects in glucan synthesis lead to reduced viability [51].

The *clc1Δ* mutant had significant defects in cell wall composition, β(1,3)glucan synthase activity, and the distribution of the β(1,3)glucan synthases. Adding sorbitol to the medium did not stabilize either Clc1p or Chc1p, and did not correct the defects in Syb1 localization detected in the 41X*HAclc1* cells after long repression times. Thus, the most plausible explanation for the rescue of the lethality of the *S. pombe clc1Δ* mutation by sorbitol is that this lethality is due to the defects in cell wall synthesis detected in the mutant.

It is possible that the synthesis of β(1,3)glucan is also regulated by the clathrin-dependent trafficking mechanisms in other fungi; however, in these organisms the compensatory mechanisms would promote chitin synthesis and the construction of a remedial cell wall that would protect cells against lysis and would allow viability in clathrin-defective mutants.

### Vesicle trafficking and cell wall synthesis in *clc1* mutants

The 41X*HAclc1* cells grown under de-repressing conditions exhibited reduced localization of glucan synthases at the cell surface, and diminished glucan synthesis. Under the same conditions, general secretion and endocytosis were not impaired; therefore, the defects in the cell wall were due to a specific alteration in the regulation of its biosynthesis by clathrin. These defects could be caused by a reduced exocytosis or by an enhanced endocytosis. A blockade in endocytosis by adding Latrunculin A to the medium or by deleting *end4^+^* stabilizes Bgs1p at the cell surface under osmotic stress conditions [31]; here, we have found that β(1,3)glucan synthase activity is greatly increased in an *end4Δ* strain grown under standard laboratory conditions, showing that this activity is regulated by endocytosis. If the main mechanism of regulation of glucan synthesis by clathrin was endocytosis, in the 41X*HAclc1* mutant the activity should be higher than in the control strain. On the contrary, we have found that the activity was lower in the mutant. Additionally, after six hours of repression the activity was further reduced in the mutant strain, while there was a significant reduction in the efficiency of general endocytosis (FM4-64 uptake) under these conditions. It is possible that a clathrin-independent endocytosis mechanism is activated in the mutant so that endocytosis of the Bgs enzymes is enhanced; this mechanism would have to be independent on End4p, since in the *end4Δ* strain the β(1,3)glucan synthase activity was higher than in the control. Furthermore, we have found strong evidence that favours the idea that cell wall defects in the conditional mutant are the consequence of specific defects in exocytosis in this strain.

*S. pombe apm1Δ* cells are sensitive to antifungal drugs that inhibit cell wall synthesis and exhibit mislocalized Bgs1p [40], [34]. We have found that in the *apm1Δ* mutant β(1,3)glucan synthase activity is lower than in the wild-type strain. The activity in the 41X*HAclc1* strain grown under de-repressing conditions was lower than in the control strain and higher than in the *apm1Δ* mutant; this result is in agreement with the observation that Syb1-GFP is localized at the cell surface in 41X*HAclc1* but not in *apm1Δ* cells, and with the hypothesis that under de-repressing conditions vesicle trafficking is not completely disturbed in the 41X*HAclc1* mutant. Under repressing conditions, the activity is further reduced, being more similar to that of the *amp1Δ* strain. Additionally, FRAP and Western blot analyses showed a delay in the secretion of the β(1,3)glucan synthases Bgs1p and Bgs4p, and the endo-β(1,3)glucanase Eng1p. In summary, we have confirmed that the rate of secretion of enzymes required for cell wall synthesis is reduced when the amount of clathrin is diminished. The works by Kita *et al*. and by Yu *et al*. [40], [34], which were directed to characterize the function of the AP-1 complex, gave some information about the relationship between the secretory machinery and cell wall synthesis. Since in *S. cerevisiae* components of the AP-1 complex but not clathrin have been found in the vesicle coat termed exomer [52], [53], the fact that the *apm1Δ* mutant exhibits a phenotype does not necessarily imply that this phenotype is due to a defect in clathrin. Our results confirm that the defects in the localization of Bgs1p detected in the *apm1Δ* mutant are indeed due to defects in the clathrin-AP1 secretion pathway.

Acid phosphatase was secreted to the medium in the 41X*HAclc1* mutant; similarly, in the *S. cerevisiae* and the *D. discoideum chc1Δ* mutants, invertase and acid phosphatase are secreted at almost wild-type levels [19], [22], [26]. Conversely, the *D. discoideum chc1Δ* strain is defective in the secretion of α-mannosidase and β-glycosidase [26]; thus, clathrin only affects the secretion of a subset of proteins. These results favour the idea that clathrin might be specifically (although probably not exclusively) required for proper trafficking of enzymes required for cell wall synthesis/remodelling. When the amount of Clc1p is reduced these enzymes would be partially mis-sorted to some secretory organelle and/or to the vacuole. This mis-sorting would not be general since the carboxypeptidase Cpy1p was not redirected to the cell surface.

In *S. cerevisiae*, two classes of secretory vesicles have been described, low-density and high-density secretory vesicles. The plasma-membrane ATPase Pma1p and the soluble secreted enzymes invertase, acid phosphatase, and exoglucanase are found in different vesicles. Clathrin heavy chain is required for the biogenesis of only one of such vesicle subpopulations [54] while a blockade in endocytosis by *END4* deletion does not affect the biogenesis of any of them [55]. Our results suggest that in *S. pombe* clathrin might be dispensable for the transport of some soluble proteins (Cpy1p and acid phosphatase), and necessary for the origin of vesicles that would transport some proteins to the cell surface, including the β-glucan synthases and exoglucanases required for cell wall synthesis and remodelling. In *S. cerevisiae*, v- and t-SNAREs are present in both vesicle subpopulations [54]; this observation is in agreement with our results showing that in the 41X*HAclc1* mutant Syb1-GFP and GFP-Psy1 are properly localized after 6 hours of repression (figure 6 B and results not shown). Since *S. cerevisiae* and *S. pombe* are distantly related organisms [56], our results are in agreement with the proposal that branching of the exocytic pathway evolved early in evolution [54].

### Clc1p and morphogenesis

*S. pombe clc1* mutants exhibited defects in morphology. In the conditional mutant grown under de-repressing conditions, a small number of cells exhibited septa that were thickened at their central region; this number increased when *clc1^+^* was repressed and was frequent in the *clc1Δ* strain. The centripetally growing fission-yeast septum usually has a slight thickening in its centre, where it closes [57]. The abnormal thickening of the septum observed in this study suggests that cell wall synthesis in the *clc1* mutant cells is not terminated by the closure of the septum but continues for some time, maybe because of biased vesicle trafficking. Abnormally thickened septa have been observed in *S. cerevisiae sla2/end4* mutants [58]; therefore, an alternative explanation for this phenotype would be a reduction in the endocytosis of some protein required for the closure of the septum or for membrane fusion. Multiseptated and branched cells, and cell aggregates were not observed in the 41X*HAclc1* cells in the absence of thiamine but were frequent in the null mutant; these results suggest that these phenotypes were produced by an accumulation of defects in cell wall synthesis after a long time of growth in the absence of clathrin. Some of these phenotypes were probably the consequence of an inefficient cell separation caused by the defects in the structure of the septa together with the defective secretion of Eng1p.

In the 41X*HAclc1* mutant grown under de-repressing conditions, it was possible to observe cells that were polarized but were bigger than the control cells and exhibited swollen tips or central area; this defect was enhanced after *clc1^+^* repression. This phenotype could be explained by a defective cell wall, unable to support the turgor pressure efficiently, as has been described for some mutants [51]. Cell lysis was only observed when the 41X*HAclc1* mutant had been grown in the presence of thiamine for more than 12 hours. In sum, a mild reduction in Clc1p causes some cell wall synthesis defects; a further reduction in the clathrin light chain enhances these defects, whose accumulation along time would produce more drastic abnormalities in this structure, resulting in severe morphological defects and in an inability to support the internal pressure.

Another possible explanation for the presence of cells with abnormal morphology in *clc1* mutants would be an alteration of the cytoskeleton, which would lead to a partial loss of polarity and the appearance of swollen cells. In the 41X*HAclc1* mutant grown in the absence of thiamine a few cells exhibited delocalized actin patches; the number of cells with this aberrant actin distribution increased after the addition of thiamine to the culture. Additionally, under repressing conditions the mutant exhibits defects in the localization of the exocyst, a tether system that collaborates with actin in the maintenance of polarity [59]. Probably, a combination of the defects in cell wall synthesis and the defects in the exocyst and the cytoskeleton contribute to the aberrant morphology of *clc1* cells.

In summary, the analysis of *S. pombe clc1* mutants, and the comparison of their phenotypes with those of *S. cerevisiae* and *D. discoideum* clathrin mutants shows that clathrin light chain shares some functions in all these unicellular organisms. However there is a relevant difference, in *S. pombe* Clc1p is essential for growth under standard laboratory conditions. According to our results, the main reasons for this difference are the relevance of Clc1p for Chc1p stability and the dependence of viability on glucan biosynthesis in the fission yeast.

The present work has established for the first time a relationship between β(1,3)glucan synthesis and clathrin-mediated secretion; also, it has demonstrated that β(1,3)glucan synthase is regulated by endocytosis under standard laboratory conditions.

## Supporting Information

Figure S1 **Acid phosphatase activity.** The activity was measured in the culture medium and cell extracts as indicated in Materials and methods. The values were normalized per ml of culture. Samples were collected from logarithmic cultures (in YES with sorbitol) when each culture reached the indicated OD_600nm_. The percentage of the activity in the medium with respect to the total activity (activity in the medium plus activity in cell extracts) was used to estimate the percentage of secretion.
(TIF)

Figure S2 **41X** ***HAclc1*** **cells do not mis-sort Cpy1p to the cell surface.** Detection of secreted Cpy1p by Western dot-blot analysis in the indicated strains grown in minimal medium without thiamine for 5 days at 28°C. The experiment cannot be performed in minimal medium with thiamine because 41X*HAclc1* cells die before 24 hours of incubation in the presence of the vitamin.
(TIF)

Table S1 **Source of the strains used in this work.**
(DOC)
