# Supplementary figures and images for: Regulation of Cell Wall Synthesis by the Clathrin Light Chain Is Essential for Viability in Schizosaccharomyces pombe
Source: PLoS One. 2013 Aug 19;8(8):e71510. doi: 10.1371/journal.pone.0071510 (PMC3747244; doi:10.1371/journal.pone.0071510)

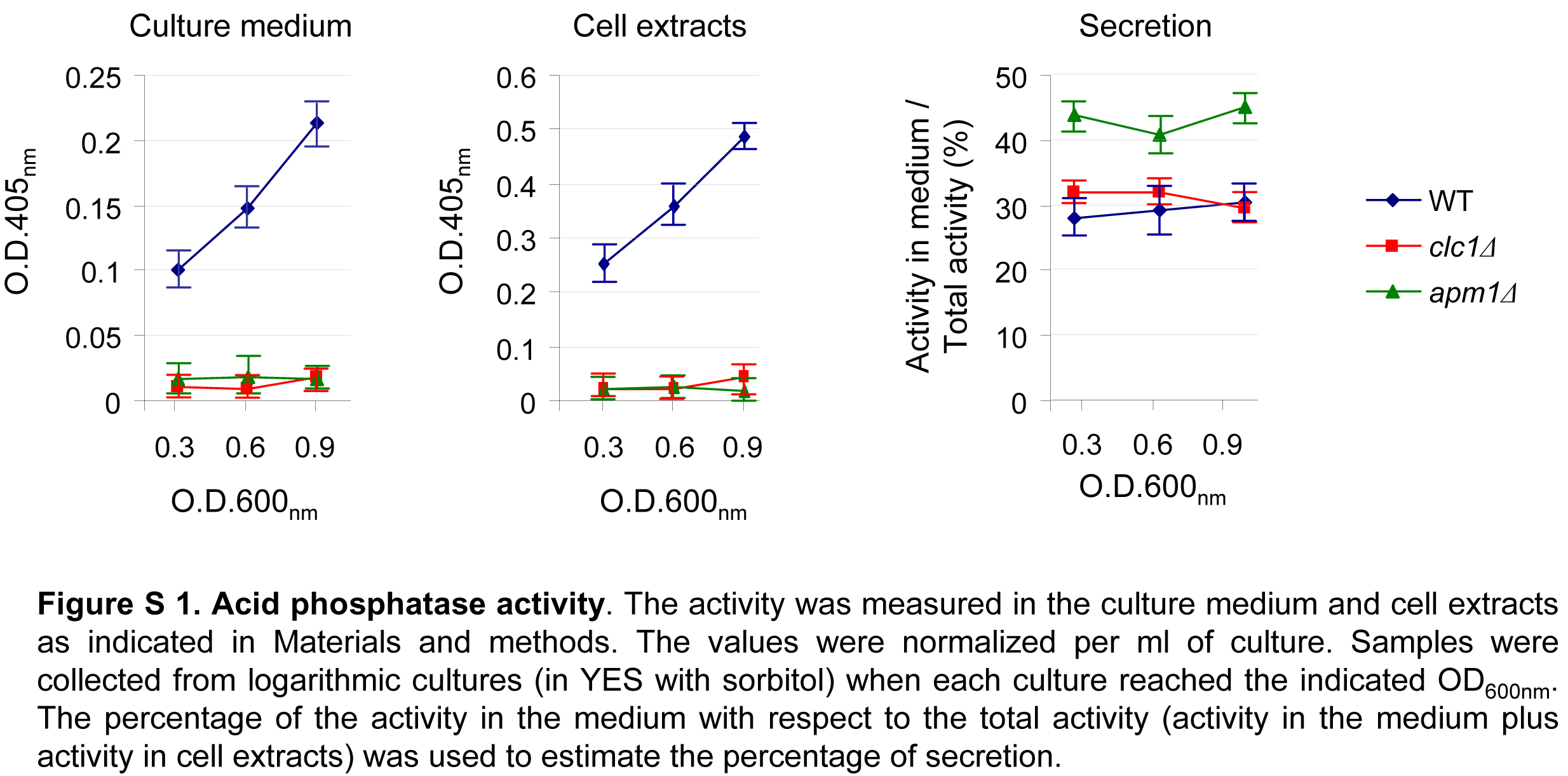

Supplement: Figure S1 — Acid phosphatase activity. The activity was measured in the culture medium and cell extracts as indicated in Materials and methods. The values were normalized per ml of culture. Samples were collected from logarithmic cultures (in YES with sorbitol) when each culture reached the indicated OD600nm. The percentage of the activity in the medium with respect to the total activity (activity in the medium plus activity in cell extracts) was used to estimate the percentage of secretion. (TIF) [file pone.0071510.s001.tif]

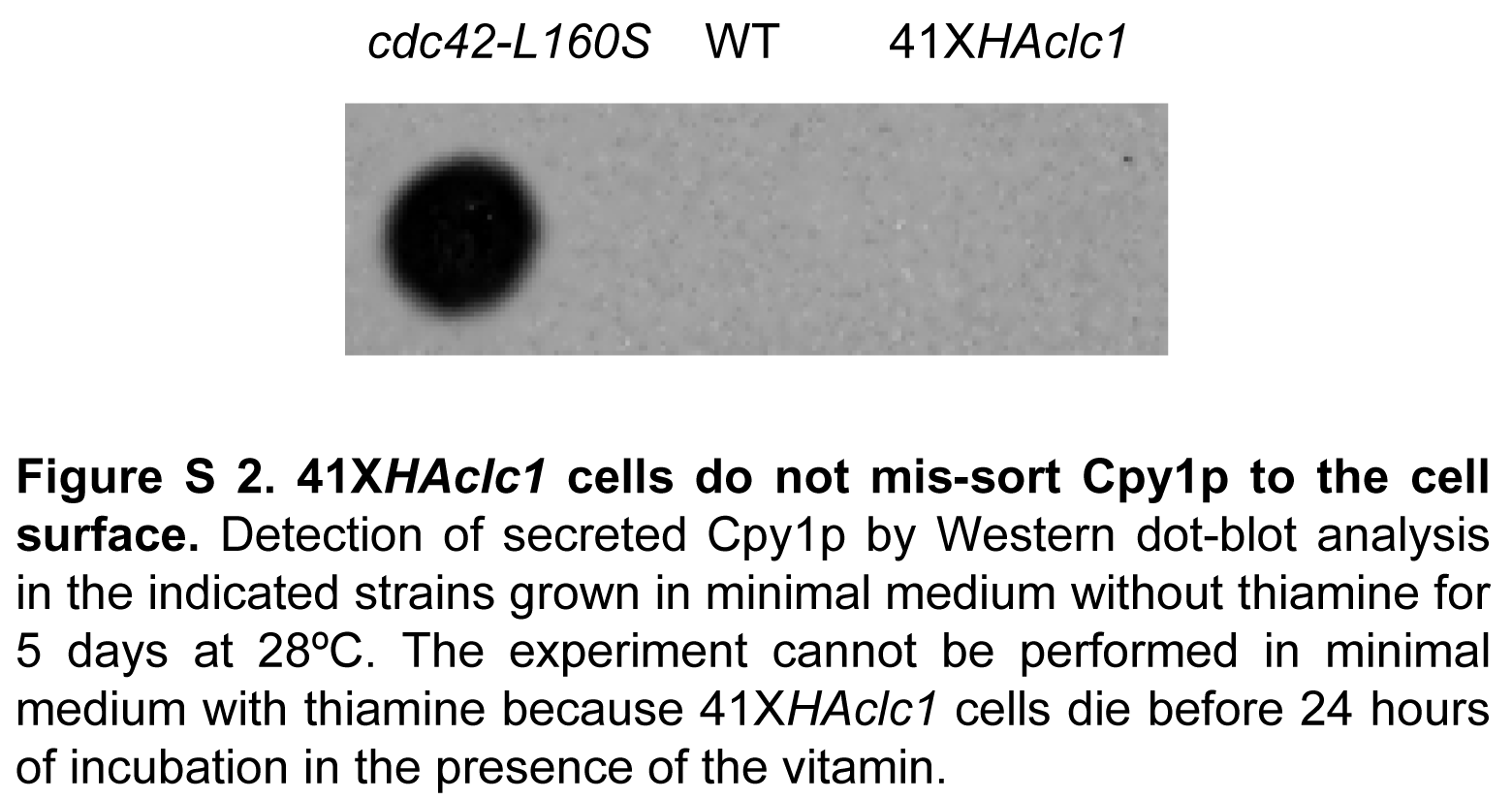

Supplement: Figure S2 — 41X HAclc1 cells do not mis-sort Cpy1p to the cell surface. Detection of secreted Cpy1p by Western dot-blot analysis in the indicated strains grown in minimal medium without thiamine for 5 days at 28°C. The experiment cannot be performed in minimal medium with thiamine because 41XHAclc1 cells die before 24 hours of incubation in the presence of the vitamin. (TIF) [file pone.0071510.s002.tif]
