# Supplementary material for: Regulation of Cell Wall Synthesis by the Clathrin Light Chain Is Essential for Viability in Schizosaccharomyces pombe
Source: PLoS One. 2013 Aug 19;8(8):e71510. doi: 10.1371/journal.pone.0071510 (PMC3747244; doi:10.1371/journal.pone.0071510)
Supplement: Table S1 — Source of the strains used in this work. (DOC) [file pone.0071510.s003.doc]

**Table S1: Source of the strains used in this work**

| **STRAIN** | **GENOTYPE** | **SOURCE** |
| --- | --- | --- |
| HVP30 | *leu1-32 his3-Δ1 ura4Δ18 ade6-M210 h^-^* | Lab. stock |
| HVP117 | *leu1-32 his3-Δ1 ura4Δ18 ade6-M216 h^+^* | Lab stock |
| HVP124 | *leu1-32/leu1-32 ura4Δ18/ura4Δ18 ade6-M210/ade6-M216 h^+^/h^-^* | Lab stock |
| HVP1221 | *sec8-GFP :ura4^+^ leu1-32 h-* | M. Balasubramanian |
| HVP1469 | *bgs4::ura4^+^ Pbgs4:GFP-Bgs4:leu1^+^ h^-^* | J.C. Ribas |
| HVP1471 | *bgs1::ura4^+^ Pbgs1:GFP-Bgs1:leu1^+^ his3-Δ1 h^-^* | J.C. Ribas |
| HVP1705 | *sla2/end4-GFP:KAN leu1-32 ura4Δ18 ade6 h-* | P. Nurse |
| HVP1711 | *end4::ura4^+^ leu1-32 h^90^* | K. Takegawa |
| HVP2031 | *sad1-GFP:KAN leu1-32 ura4D18 ade6 h^+^* | P. Perez |
| HVP2092 | *apm1::ura4^+^ leu1-32 h^-^* | T. Kuno |
| HVP2093 | *clc1^+^/clc1::KAN leu1-32/leu1-32 h^+^/h^-^* | this work |
| HVP2032 | *GFP-psy1:leu1^+^ h^90^* | Yeast Genetic Resource Center (Japan) |
| HVP2215 | *clc1::KAN leu1-32 h^+^* | This work |
| HVP2273 | *GFP:syb1::KAN leu1-32 h^-^* | Y. Sánchez |
| HVP2364 | *clc1::KAN leu1-32 ura4Δ14 ade6* with pAL+*GFP-Bgs1* | This work |
| HVP2365 | *clc1::KAN leu1-32 ura4Δ14 ade6* with pAL+*GFP-Bgs3* | This work |
| HVP2366 | *clc1::KAN leu1-32 ura4Δ14 ade6* with pAL+*GFP-Bgs4* | This work |
| HVP2388 | *chc1-GFP:leu1^+^ leu1-32 ura4Δ18 ade6 h^90^* | This work |
| HVP2466 | *41XHAclc1:KAN leu1-32 ura4Δ18 h^+^* | This work |
| HVP2468 | *41XHAclc1:KAN leu1-32 ura4Δ18 h^-^* | This work |
| HVP2592 | *41XHAclc1:KAN syb1-GFP:KAN* | This work |
| HVP2726 | *41XHAclc1:KAN with pAU+eng1-GFP leu1-32 ura4D14 h^+^* | This work |
| HVP2777 | *41XHAclc1:KAN chc1-GFP:leu1^+^ leu1-32 ura4D18* | This work |
| HVP2779 | *41XHAclc1:KAN bgs1::ura4^+^ GFP-Bgs1:leu1^+^* | This work |
| HVP2783 | *41XHAclc1:KAN bgs1::ura4^+^ GFP-Bgs41:leu1^+^* | This work |
| HVP2784 | *41XHAclc1:KAN apl3-GFP:leu1^+^* | This work |
| HVP2810 | *apm1-GFP:leu chc1-mcherry2476:ura4* | This work |
| HVP2835 | *rho3::ura4^+^ leu1-32 ura4Δ18 ade6 h-* | M. Balasubramanian |
| HVP2876 | *41XHAclc1:KAN sec8-GFP:ura4^+^* | This work |
| HVP2877 | *41XHAclc1:KAN end4/Sla2-GFP:KAN* | This work |
| HVP3008 | *41XHAclc1:KAN GFP-Psy1:leu1^+^* | This work |
| HVP3054 | *clc1-GFP:ura4^+^ TomatoRFP-Bgs1:leu1^+^* | This work |
| HVP3328 | *41XHAclc1:KAN apm1-GFP:leu1^+^ chc1-mCherry:ura4^+^* | This work |
| HVP3375 | *HA-Clc1:leu1+ leu1-32 ura4Δ18 ade6-M216 his3Δ1 h^+^* | This work |
| PPG6521 | *leu1-32 ura4-D18 HA-cdc42L160S:ura4^+^ h^-^* | P. Pérez |
| HVP3469 | *clc1-Cherry:leu1+ with pAU+eng1-GFP* | This work |
| HVP3471 | *leu1-32 his3-Δ1 ura4Δ18 ade6-M216 h^+^ with pAU* | This work |
| HVP3472 | *leu1-32 his3-Δ1 ura4Δ18 ade6-M216 h^+^ with pAU+chc1^+^* | This work |
| HVP3473 | *41XHAclc1:KAN leu1-32 his3-Δ1 ura4Δ18 ade6-M216 h^+^ with pAU* | This work |
| HVP3475 | *41XHAclc1:KAN leu1-32 his3-Δ1 ura4Δ18 ade6-M216 h^+^ with pAU+chc1^+^* | This work |
